# Supplementary material for: CEP44 ensures the formation of bona fide centriole wall, a requirement for the centriole-to-centrosome conversion
Source: Nat Commun. 2020 Feb 14;11:903. doi: 10.1038/s41467-020-14767-2 (PMC7021698; doi:10.1038/s41467-020-14767-2)

CEP44 ensures the formation of *bona fide* centriole wall, a requirement for the centriole-to-centrosome conversion

Atorino et al.

Supplementary figure 1. CEP44 drives dC maturation into centrosomes. Related to figure 1

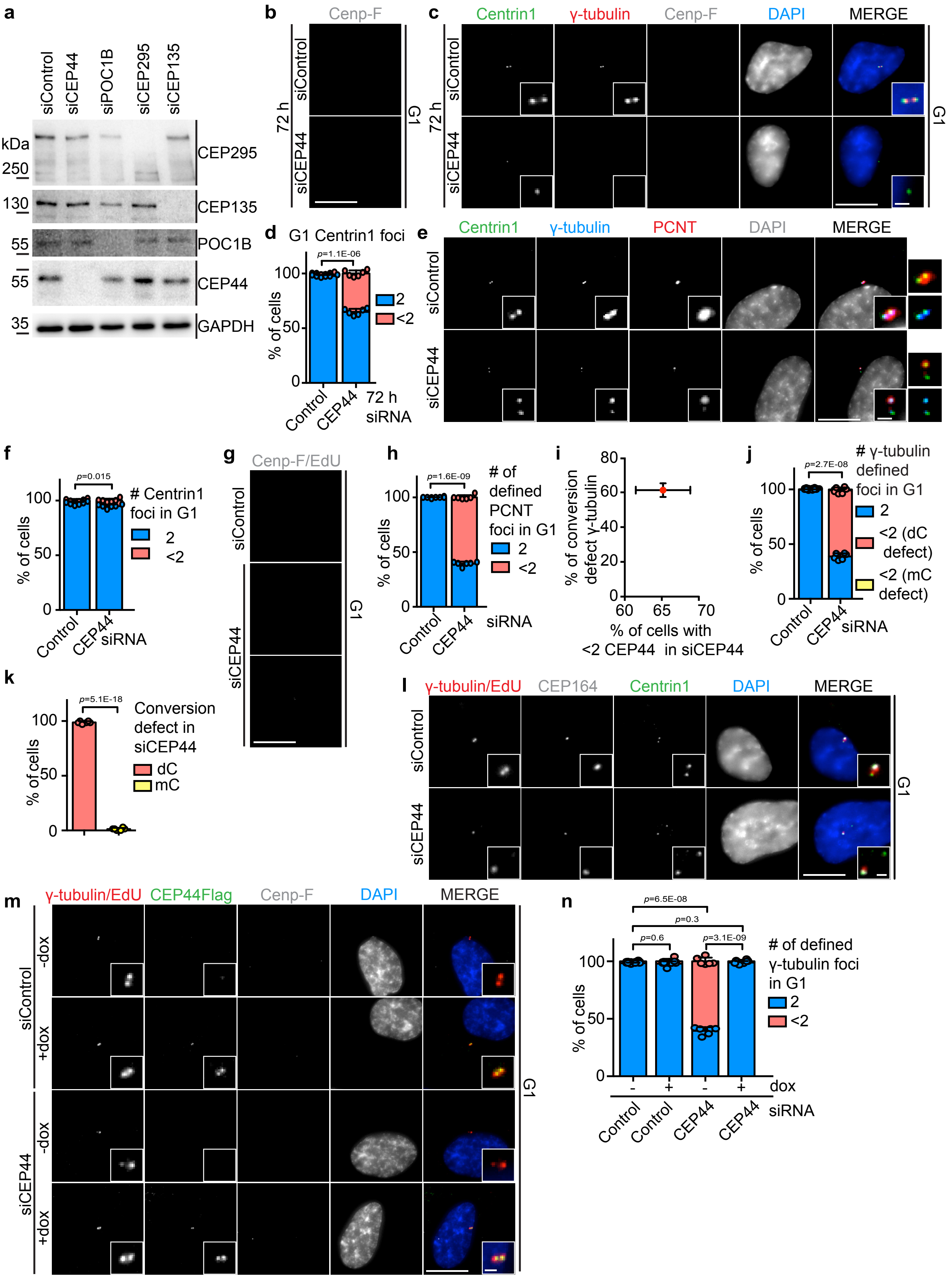

Supplementary figure 1. CEP44 drives dC maturation into centrosomes. Related to figure 1. (a) IB of RPE1 cell lysates depleted with either siControl, siCEP44, siPOC1B, siCEP295 or CEP135 to test the depletion specificity and efficiency. Note, that in the siPOC1B sample less protein was loaded than in the control because POC1B depletion inhibits cell proliferation[38]. Samples were derived from the same experiment and the blots were processed in parallel. (b) Cenp-F negative G1 cells from Fig. 1b. (c) 72 h depletion of CEP44 generated loss of centrioles (centrin1) in G1 cells. (d) Quantification of (c). 35.0±2.8% of cells with <2 centrin1 foci. (e) IF showing that although the centriole number remained unaffected in siCEP44 depleted cells (centrin1 staining), the centrioles in siCEP44 samples had a lower PCM (γ-tubulin and PCNT) recruitment efficiency. (f) Centrin1 foci number quantification of (e). (g) Cenp-F/EdU negative stain of G1 cells from Fig. 1d. (h) 60.6±1.9% of G1 siCEP44 cells showed <2 PCNT foci. (i) Correlation between the CEP44 loss (65.1±7.3% of the cells) and the conversion defect (60.7±4.2 G1 cells with <2 γ-tubulin defined foci) upon siCEP44. (j, k) Association of the CCC defect to mC and dC. In siCEP44 sample 60.5±2.8% of the dCs showed CCC defect and the rest of the defective centrosomes were mCs 0.8±0.7%. Meaning that of the defective centrosomes, the 98.8±1.1% were dC (k). (l) Conversion defect in siCEP44 cells correlated with centrioles that lacked the mC marker CEP164. (m) RPE1 stable cell line with integrated exogenous *CEP44-Flag* cDNA that carries neutral point mutations in the coding region to make the mRNA siRNA-resistant. *CEP44* expression was under control of the TetOn system. Even low expression (2 ng/ml of doxycycline) of the recombinant could rescue the conversion defect in G1 cells. (n) Quantification of (m). The loss of γ-tubulin in the CEP44 depletion sample (69.4±3.2%) was completely rescue by expression of *CEP44-Flag*. (b, c, e, g, l, m, scale bars: 10 μm,

magnification scale bars: 1 μm; d, f, h, i, j, k and n data presented as mean±s.d., all statistics were derived from two-tail unpaired t-test analysis of n=6 biologically independent experiments and source data are provided as a Source Data file).

Supplementary figure 2. *CEP44* KO clones resemble CEP44 siRNA CCC defect. Related to figure 1

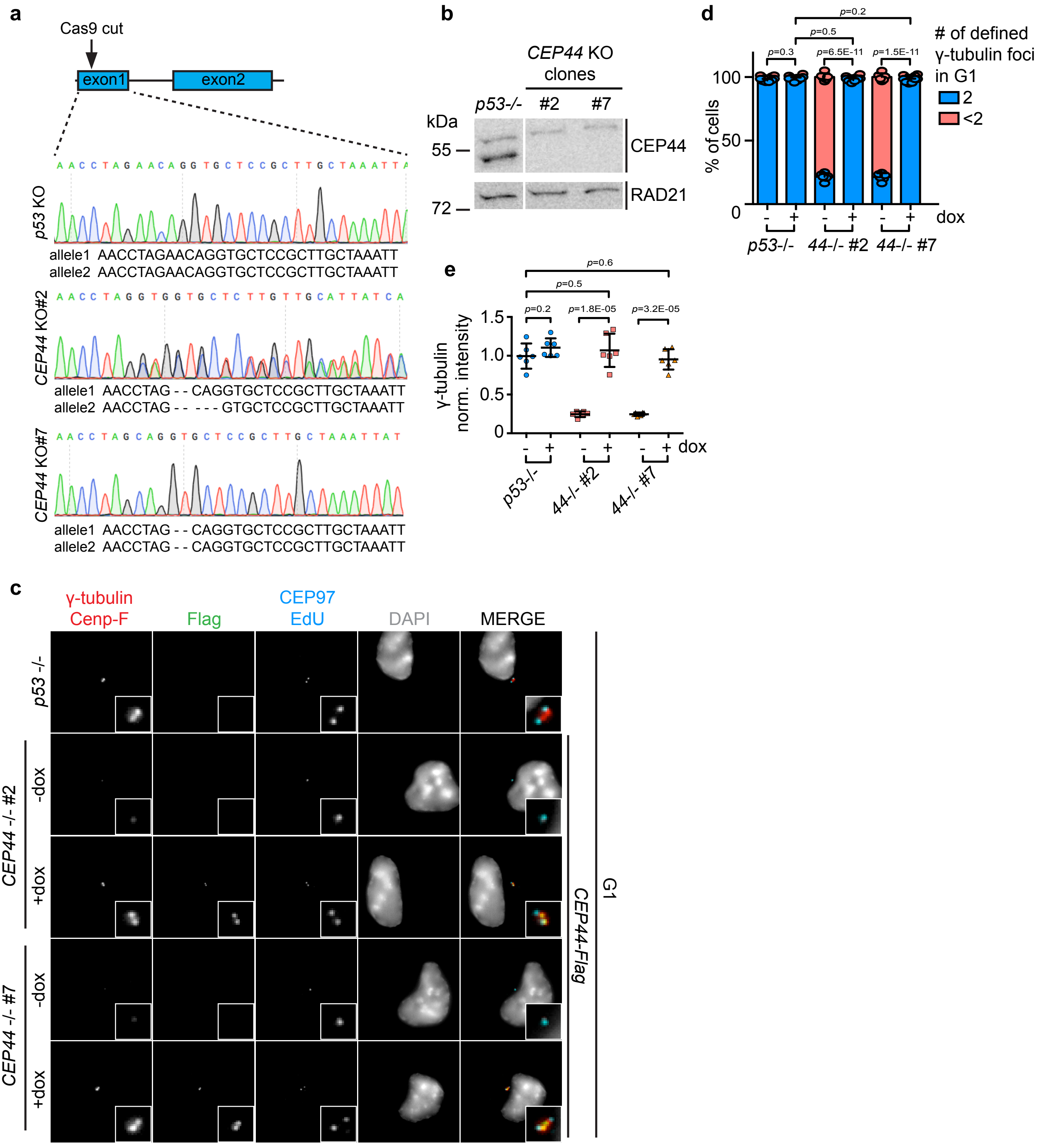

Supplementary figure 2. *CEP44* KO clones resemble CEP44 siRNA CCC defect. Related to figure 1. (a, top) Scheme of CRISPR/Cas9 cut in the *CEP44* gene. (a, bottom) *CEP44* genotype sequences of the *p53* KO RPE1 cell line from which *CEP44* KOs were originated and of two biological independently clones for *CEP44* KO. (b) IB of the lysates of the *p53* KO strain and of the two *CEP44* KO clones. (c) IF of RPE1 *p53* KO cell line and *CEP44* KO clones with integrated exogenous *CEP44-Flag* cDNA. *CEP44* expression was under control of the TetOn system. Expression of the recombinant *CEP44-Flag* (2 ng/ml of doxycycline) could rescue the conversion defect generated by the KO of *CEP44*. (d and e) Quantifications of (c). (d) In absence of CEP44, the two clones lost the centrosomes (judged by γ-tubulin defined foci) in 78.5±2.9% (clone #2) and 78.6±2.9% (clone #7) of G1 cells. This CCC phenotype was completely rescued upon CEP44-Flag doxycycline induction. (e) Normalized intensity measurements of γ-tubulin on centrosomes present in all the conditions of (c) Both *CEP44* KO clones showed a reduction of γ-tubulin intensity of 75%. (c, scale bar: 10 μm, magnification scale bar: 1 μm; d and e data are presented as mean±s.d., all statistics were derived from two-tail unpaired t-test analysis of n=6 biologically independent experiments and source data are provided as a Source Data file).

Supplementary figure 3. CEP44 loss affects the recruitment of CEP135, CEP152 and CEP192 to dCs. Related to figure 2

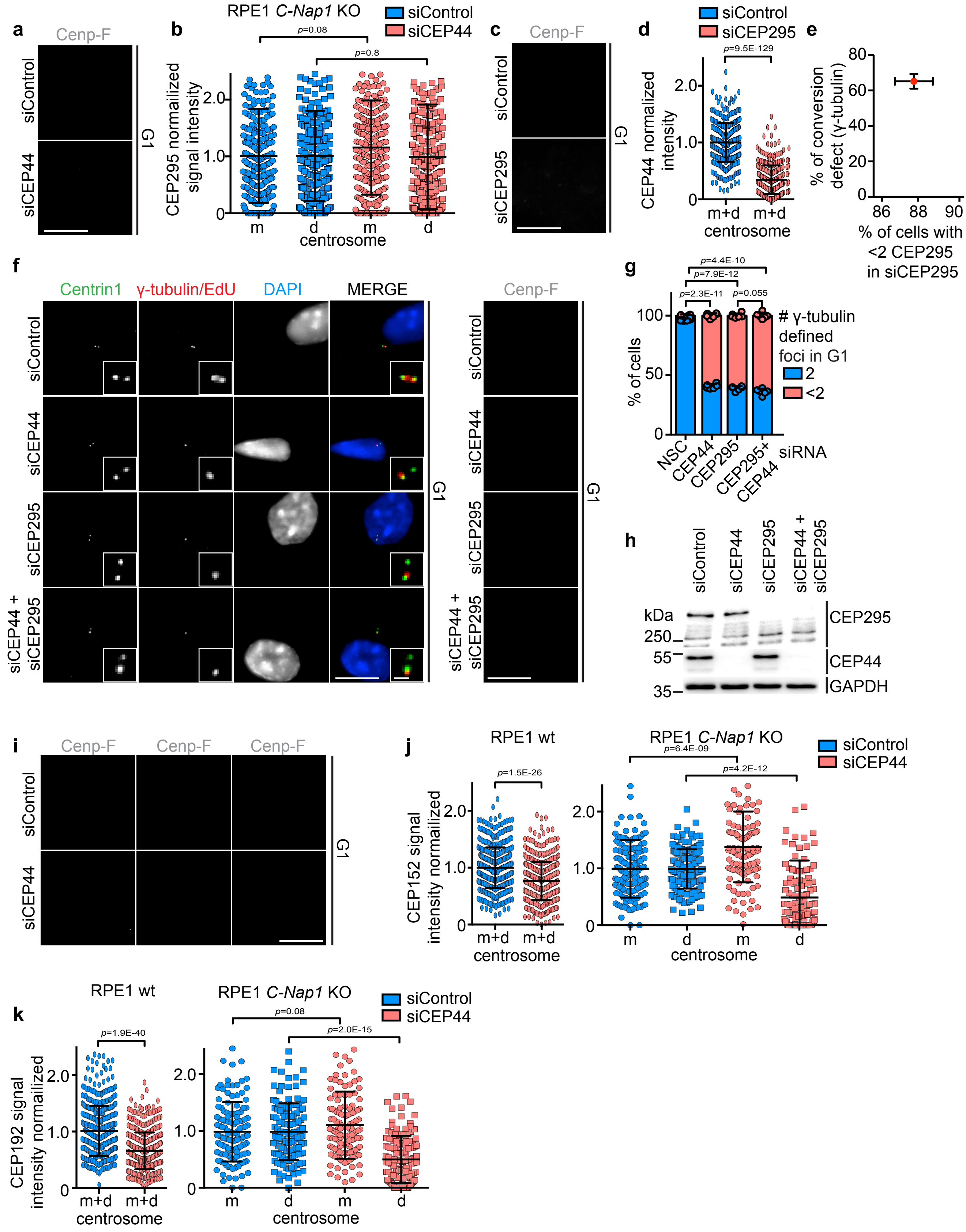

Supplementary figure 3. CEP44 loss affects the recruitment of CEP135, CEP152 and CEP192 to dCs. Related to figure 2. (a) Cenp-F stain from Fig. 2a. (b) CEP295 normalized intensities on mother or daughter centrosomes in G1 *C-Nap1* KO. n=270 (siControl) and n=264 (siCEP44) cells over 6 biologically independent experiments. (c) Cenp-F stain from Fig. 2c. (d) Normalized reduction of CEP44 signal (to 0.7±0.2) in RPE1 cells upon siCEP295 (Fig. 2c). n=386 (siControl) and n=378 (siCEP295) cells over 6 biologically independent experiments. (e) Correlation between CEP295 loss (87.1±2.1% of the cells) and the conversion defect (66.5±4.2 G1 cells with <2 γ-tubulin defined foci) upon siCEP295. n=6 biologically independent experiments. (f) Loss of two defined γ-tubulin (g, siCEP44 40.6±2.0%, siCEP295 38.9±1.9 and siCEP295+siCEP44 36.1±2.5 of G1 cells) in the cells treated with siCEP295 and siCEP44 or siCEP295+siCEP44. (g) Quantification of (f). n=6 biologically independent experiments. (h) IB of samples treated with the indicated siRNAs. Samples were derived from the same experiment and the blots were processed in parallel. (i) Cenp-F stain Fig. 2e (left panel), Fig. 2g (middle) and Fig. 2i (right). (j and k) Left panels show the reduction of normalized signal intensities for CEP152 or CEP192 measured at the mother and daughter centrosomes together in RPE1 G1 cells treated with siControl or siCEP44. CEP152 reduction to 0.8±0.3 and CEP192 to 0.6±0.3. (j, left) n=569 (siControl) and n=513 (siCEP44) and (k, left) n=479 (siControl) and n=475 (siCEP44) cells, all over 6 biologically independent experiments. (j and k, right panels) Normalized intensities of CEP152 and CEP192 measured at the mother or daughter centrosomes in G1 RPE1 *C-Nap1* KO cells treated with siControl or siCEP44. In both cases the intensity was reduced at least on the daughter centrosomes in the siCEP44. Normalized reduction of CEP152 signal on daughter centrosome to 0.4±0.7 and CEP192 to 0.5±0.4. (j, right) n=165 (siControl) and n=119

(siCEP44) and (k, right) n=132 (siControl) and n=132 (siCEP44) cells, all over 3 biologically independent experiments. (a, c, f, i, scale bars: 10 μm, magnification scale bars: 1 μm; b, d, e, g, j and k data presented as mean±s.d.; all statistics derived from two-tail unpaired t-test analysis of the specified “n” and source data provided as a Source Data file).

Supplementary figure 4. CEP135 localizes to the dCs later than CEP295, and it did not affect CCC
Related to figure 2

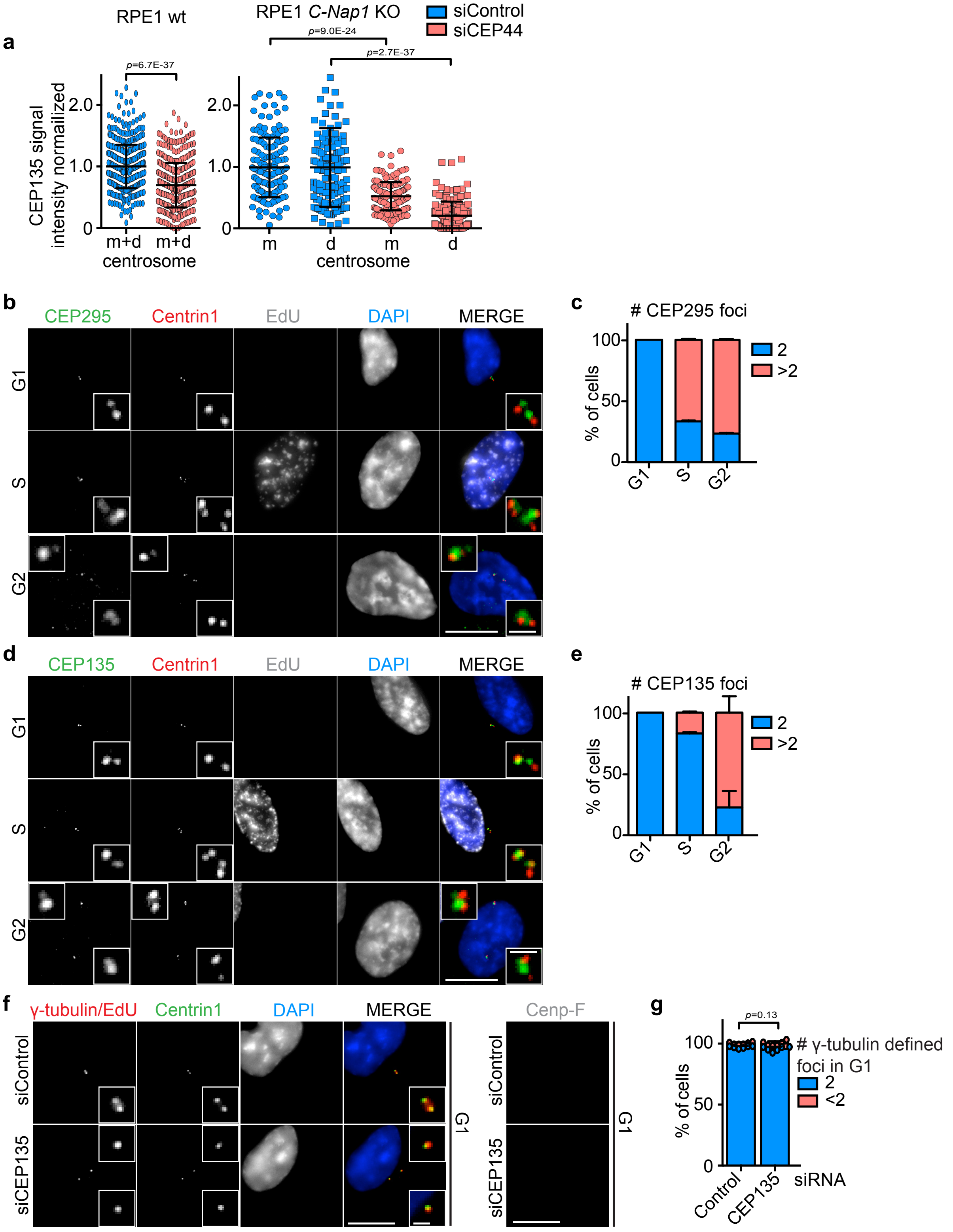

Supplementary figure 4. CEP135 localizes to the dCs later than CEP295 and it did not affect CCC. Related to figure 2. (a) The left panel shows the reduction of normalized signal intensities for CEP135 measured at the mother and daughter centrosomes together in RPE1 wild type G1 cells treated with siControl or siCEP44. CEP135 reduction to 0.7±0.4. Statistics were derived from two-tail unpaired t-test analysis of n=537 (siControl) and n=504 (siCEP44) over 6 biologically independent experiments. (a, right) shows normalized intensities of CEP135 measured separately at either the mother or the daughter centrosomes in G1 RPE1 *C-Nap1* KO cells treated with siControl or siCEP44. The normalized intensity was reduced at least on the daughter centrosomes in the siCEP44 sample. Normalized reduction of CEP135 signal on daughter centrosome to 0.2±0.2. Statistics were derived from two-tail unpaired t-test analysis of n=147 (siControl) and n=157 (siCEP44) over 3 biologically independent experiments. (b) IF of cycling RPE1 cells (as in Fig. 1a) showing that CEP295 binds to the new dCs during S phase (c, 66.2±0.7% of S phase cells with >4 CEP295 foci). (d) IF of cycling RPE1 cells showing that CEP135 stars to binds to the new dCs mostly in the G2 (e, 77.2±0.13.4% of G2 phase cells with >4 CEP295 foci vs. 17.0±1.0% in S phase). (c and e) Quantifications of (b and d). In both cases n>180 cells. (f) No detectable γ-tubulin recruitment defect in G1 cells of siCEP135 sample. (g) Quantification of (f). Statistics derived from two-tail unpaired t-test analysis of n=6 biologically independent experiments. Source data of a, c, e, and g are provided as a Source Data file. (b, d, f, scale bars: 10 μm, magnification scale bars: 1 μm; a, c, e and g data are presented as mean±s.d.).

Supplementary figure 5. Recruitment of POC1B to the centriole depends on the full lenght CEP44. Related to figure 3.

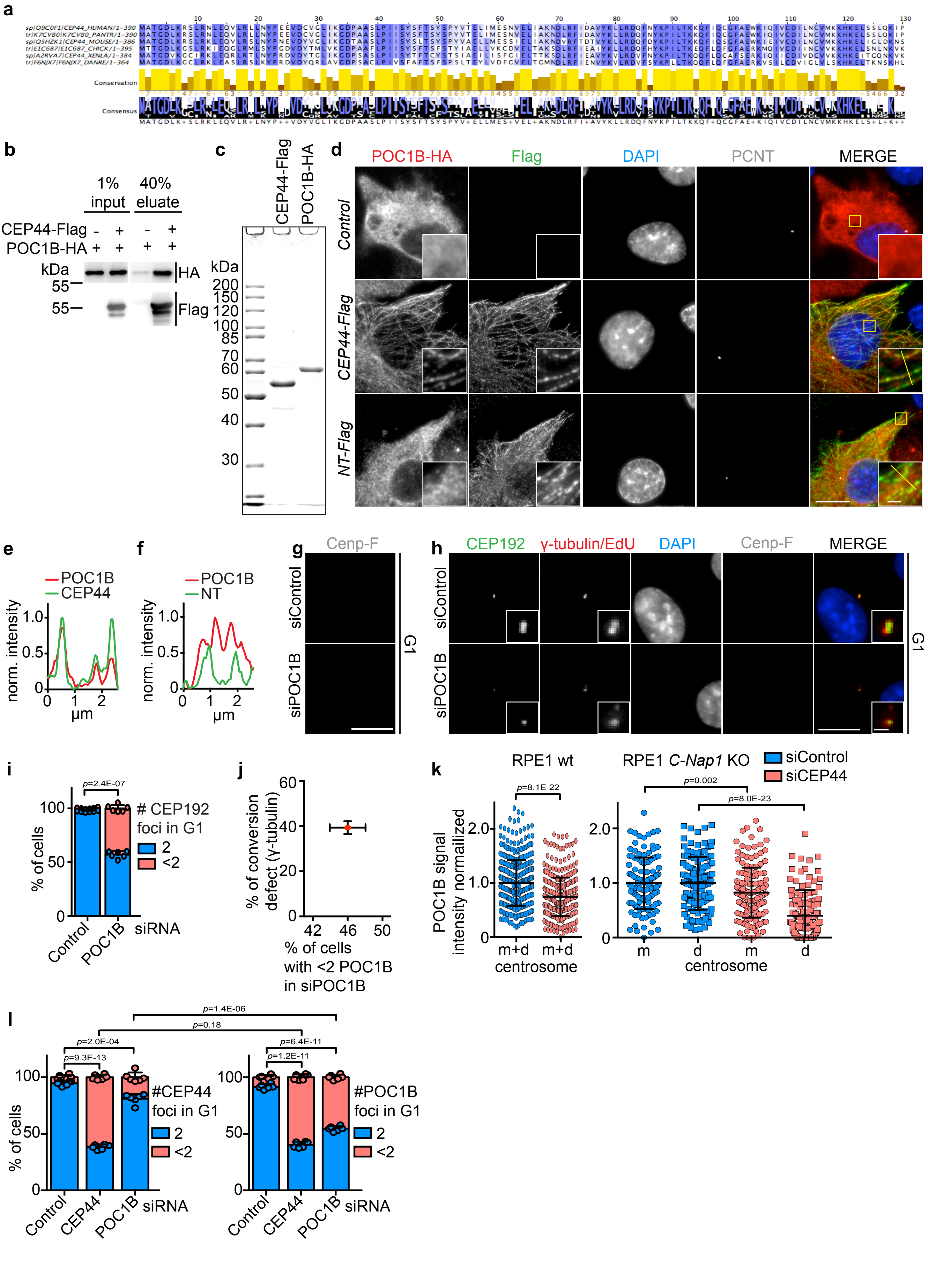

Supplementary figure 5. Recruitment of POC1B to the centriole depends on the full length CEP44. Related to figure 3. (a) Alignment of N-terminal CEP44 protein sequence of different species (human, chimpanzee, mouse, chicken, frog, zebrafish) showing strong aa conservation. (b) IB of Coomassie Blue stained gel of Fig. 3b. (c) Coomassie Blue stained gel of purified recombinant proteins used for CEP44-POC1B *in vitro* binding in (b) and in Fig. 3b. (b and c) Molecular weight markers indicated. (d) IF of RPE1 cells in which POC1B-HA was co-expressed with CEP44-Flag or NT-Flag. Overexpressed POC1B shifted its localization from the cytosol (upper panel) to MTs when the CEP44 was co-expressed (middle panel), co-localizing with it (e). This was not the case for the NT-fragment (f), confirming (Fig. 3e). (e and f) Line profiles of CEP44 and POC1B co-localization. The scanned lines indicated in the enlargements of (d). (g) Cenp-F negative stain of G1 cells from Fig. 3f. (h and i) Delocalization of CEP192 upon siPOC1B. (i) In siPOC1B G1 cells, 42.9±3.2% of the cells showed <2 CEP192 foci. n=6 biologically independent experiments. (j) Correlation between the % of G1 cells in which POC1B was efficiently depleted (45.5±2.3%) and in which the CCC defect appeared upon siPOC1B (39.2±2.8%). n=6 biologically independent experiments. (k) The left panel show reduced normalized signal intensities of POC1B at the mother and daughter centrosome together in RPE1 wt siControl and siCEP44 G1. POC1B intensity was reduced to 0.7±0.4. n=455 (siControl) and n=428 (siCEP44) over 6 biologically independent experiments. (k, right) Normalized intensities of POC1B measured separately at the mother or daughter centrosome in siControl or siCEP44 G1 RPE1 *C-Nap-1* KO cells. Intensity of POC1B was reduced at least on the daughter centrosome to 0.4±0.5 in siCEP44. n=134 (siControl) and n=152 (siCEP44) over 3 biologically independent experiments. (l) Correlation of the loss of either CEP44 or POC1B in response to siRNAs depletion

against either of them from (Fig. 3i and j). n=6 biologically independent experiments. (d, g, h, scale bars: 10 μm, magnification scale bars: 1 μm; i, j, k and l data presented as mean±s.d., all statistics derived from two-tail unpaired t-test analysis of the specified “n”) and source data provided as a Source Data file.

Supplementary figure 6. The NT-CEP44 can partially rescue POC1B recruitment to the centriole. Related to figure 3.

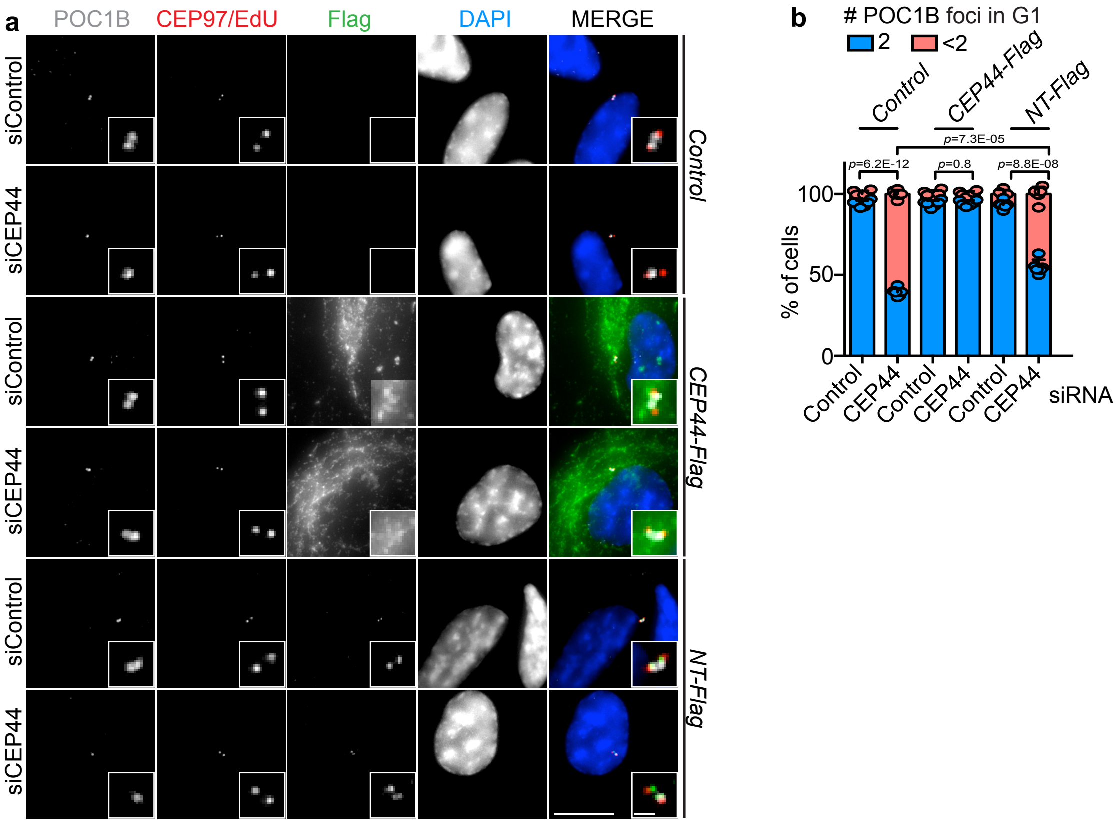

Supplementary figure 6. The NT-CEP44 can partially rescue POC1B recruitment to the centriole. Related to figure 3. (a-b) POC1B upon siCEP44 loss (60.6±2.5% of cells with <2 POC1B foci) was completely rescued by expression of siRNA resistant *CEP44*-Flag, but not completely with the expression of the NT *CEP44* (45.0±4.4% of cells with <2 POC1B foci). (b) Data are presented as mean±s.d. and statistics were derived from two-tail unpaired t-test analysis of n=6 biologically independent experiments and source data are provided as a Source Data file. (a, scale bars: 10 μm, magnification scale bars: 1 μm).

Supplementary figure 7. POC1B, differently from POC1A, generates CCC defects. Related to figure 3 Related to figure 3

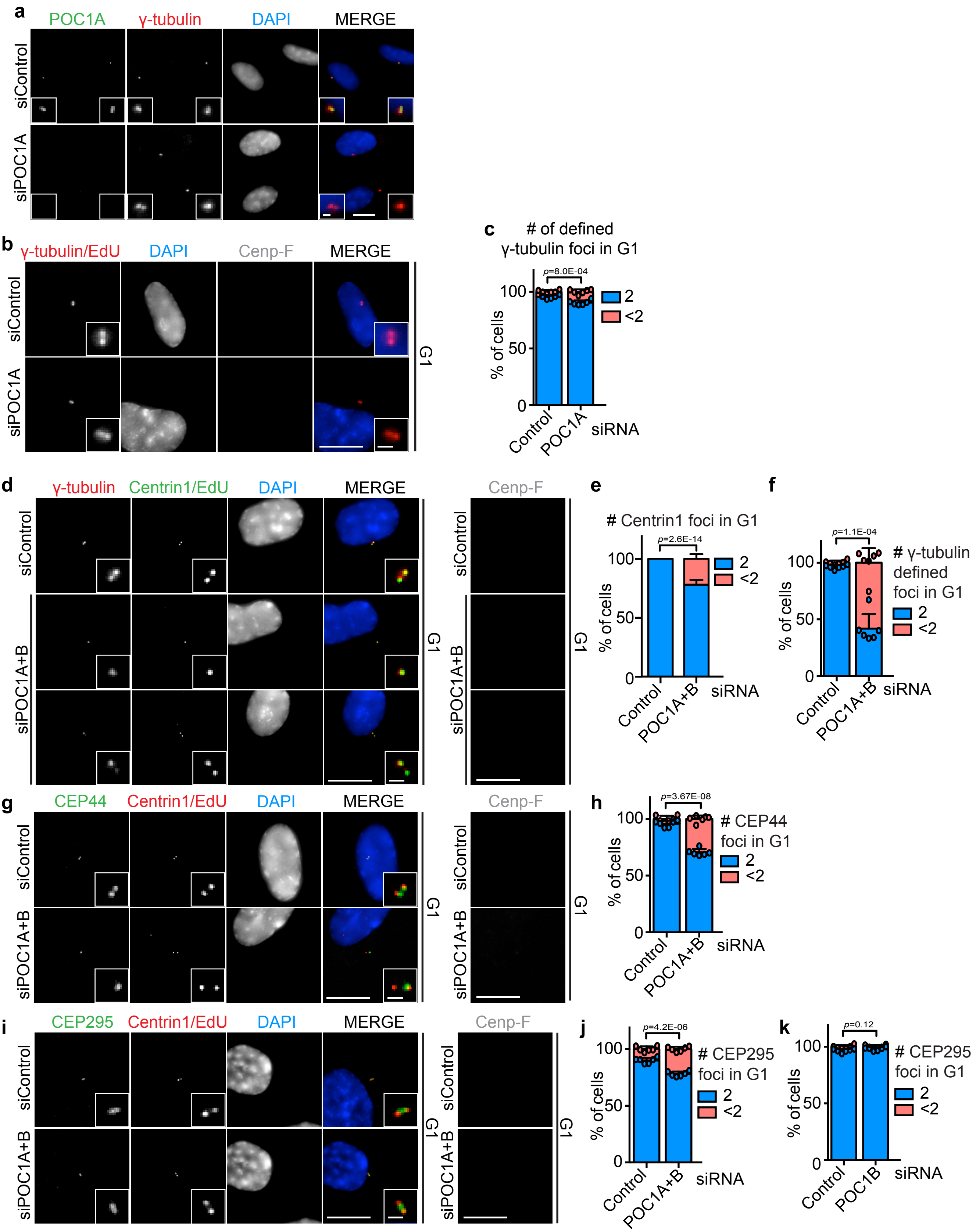

Supplementary figure 7. POC1B, differently from POC1A, generates CCC defects. Related to figure 3. (a) POC1A signal disappeared from centrosomes upon POC1A siRNA treatment. (b) G1 POC1A depleted cells barely showed a defect in recruitment of γ-tubulin. (c) Quantification of (b). A low number of G1 cells (9.6±2.0%) showed <2 γ-tubulin. (d) G1 cells in which POC1A+POC1B were co-depleted, showed centriole duplication defect (detected by centrin1) and a strong CCC defect (judged by γ-tubulin). (e) Quantification of the duplication defect (21.9±4.0% of the cells) from (d). Statistics were derived from two-tail unpaired t-test analysis of n=18 biologically independent experiments. (f) Quantification of the CCC defect (58.2±12.8% of the cells) from (d). (g) siPOC1A+siPOC1B co-depletion delocalized CEP44 from the centrioles similarly to siPOC1B in Fig. 3j. (h) Quantification of (g), in which 29.5±3.0% of the cells lost CEP44 upon siPOC1A+B. (i and j) siPOC1A+siPOC1B delocalized CEP295 from the centrioles differently from siPOC1B (k). (j) Quantification of (i), in which 21.9±2.1% of the cells lost CEP295 upon siPOC1A+B. (k) Depletion of only POC1B via siRNA did not delocalized CEP295. (a, b, d, g, i, scale bars: 10 μm, magnification scale bars: 1 μm; c, e, f, h, j and k data are presented as mean±s.d., c, f, h, j and k statistics were derived from two-tail unpaired t-test analysis of n=6 biologically independent experiments and c, e, f, h, j and k source data are provided as a Source Data file).

Supplementary figure 8. The N-terminal CEP44 region contains a conserved domain necessary for protein function. Related to figure 4

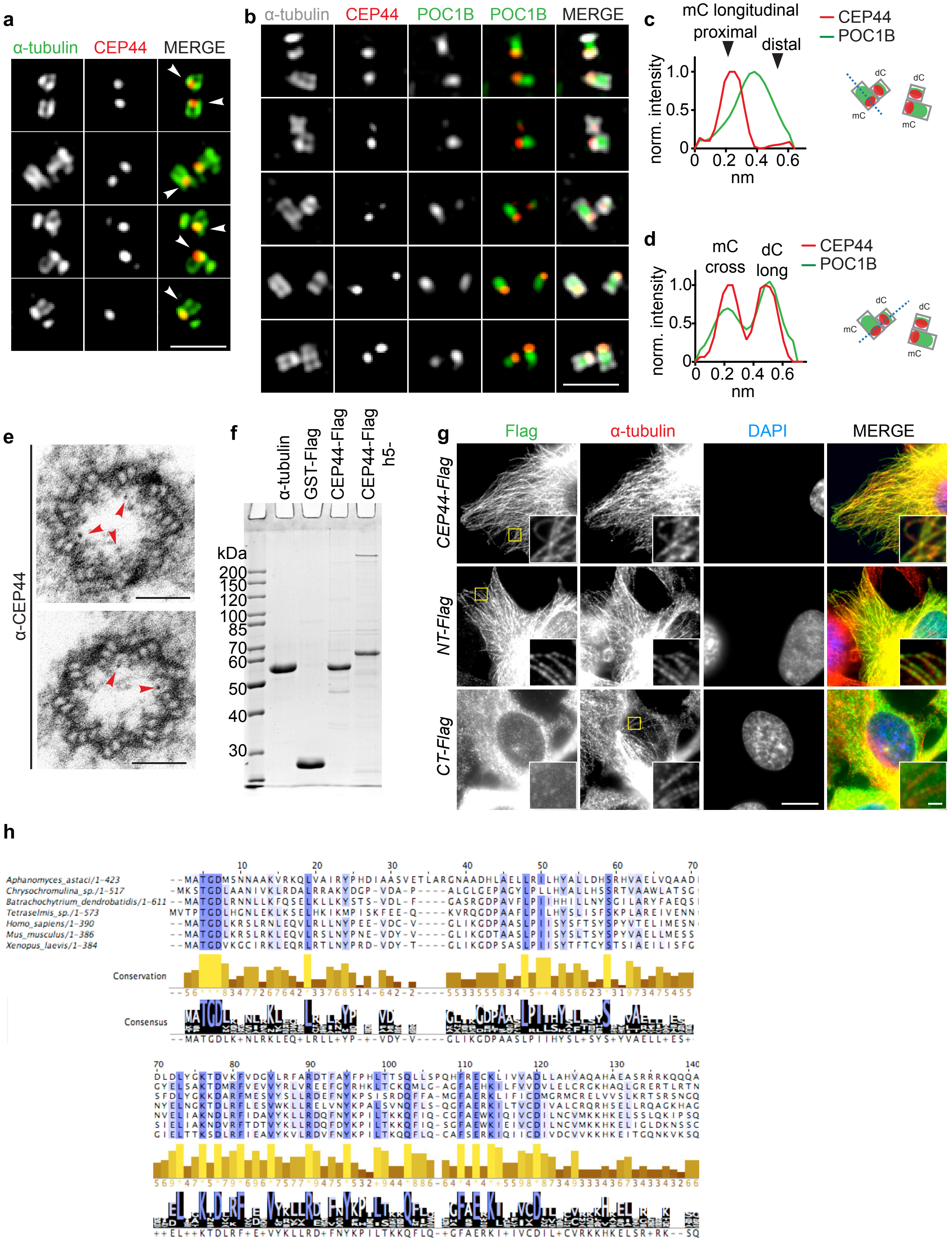

Supplementary figure 8. The N-terminal CEP44 region contains a conserved domain necessary for protein function. Related to figure 4. (a) 2D-SIM images of CEP44 localizing in the lumen of centriole pairs (α-tubulin). (b) 2D-SIM images of the complex CEP44-POC1B localizing in the lumen of centriole pairs (α-tubulin). (c) Longitudinal intensity plots profiles of mC in Fig. 4d. CEP44 signal partially co-localized with POC1B one in the mC lumen (as in Supplementary Fig. 7b). (d) Intensity plots profiles of mC (cross-section) and dC (longitudinal section) in Fig. 4d. CEP44 signal co-localized with POC1B both on the mC cross-section and dC longitudinal section. (e) Immuno-gold labelling of endogenous CEP44 in purified centrosomes. Red arrows indicate 10 nm gold particles. (f) Coomassie Blue stained gel of purified recombinant proteins used in the MT-binding assay in Fig. 4h. Molecular weight markers are indicted. (g) IF of samples overexpressing (1 μg/ml of doxycycline) the recombinant CEP44-Flag, N-terminal half (NT-Flag) and C-terminal half (CT-Flag) of the protein in RPE1 cells. While the CEP44-Flag and the NT-Flag recombinant proteins decorated the MTs (α-tubulin), the CT-Flag did not. (h) Alignment between the protein sequences of CEP44 N-terminal region from different organisms (unicellular and multicellular organisms: *Aphanomyces astaci*, *Chrysocromulina sp.*, *Batrachochytrium dentrobatidis*, *Tetraselmis sp.*, *Homo sapiens*, *Mus musculus* and *Xenopus laevis*). The alignment shows a high degree of conservation of this region. (a, b, scale bars: 1 μm; e, scale bars: 100 nm; g, scale bar: 10 μm, magnification scale bar: 1 μm).

Supplementary figure 9. Mutations in CEP44 MT-binding domain generate a dominant negative dC conversion phenotype. Related to figure 4

a

	Mutations
h1-	K7D, R8E, R11E, R18E
h5-	K68D, R72E,K79D, R82D, K88D
h1 fold stop	L13P
h3 fold stop	I43P
h4 fold stop	I57P
h6 fold stop	L113P
h1 side inter	Y22A
h3/4 side inter	Y45A, Y47A, Y50A, Y53A
h5 side inter	F73A, F78A, F85A, Y87A
h6 side inter	F96A, F101A

b

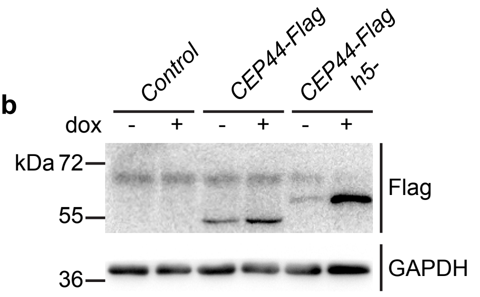

c

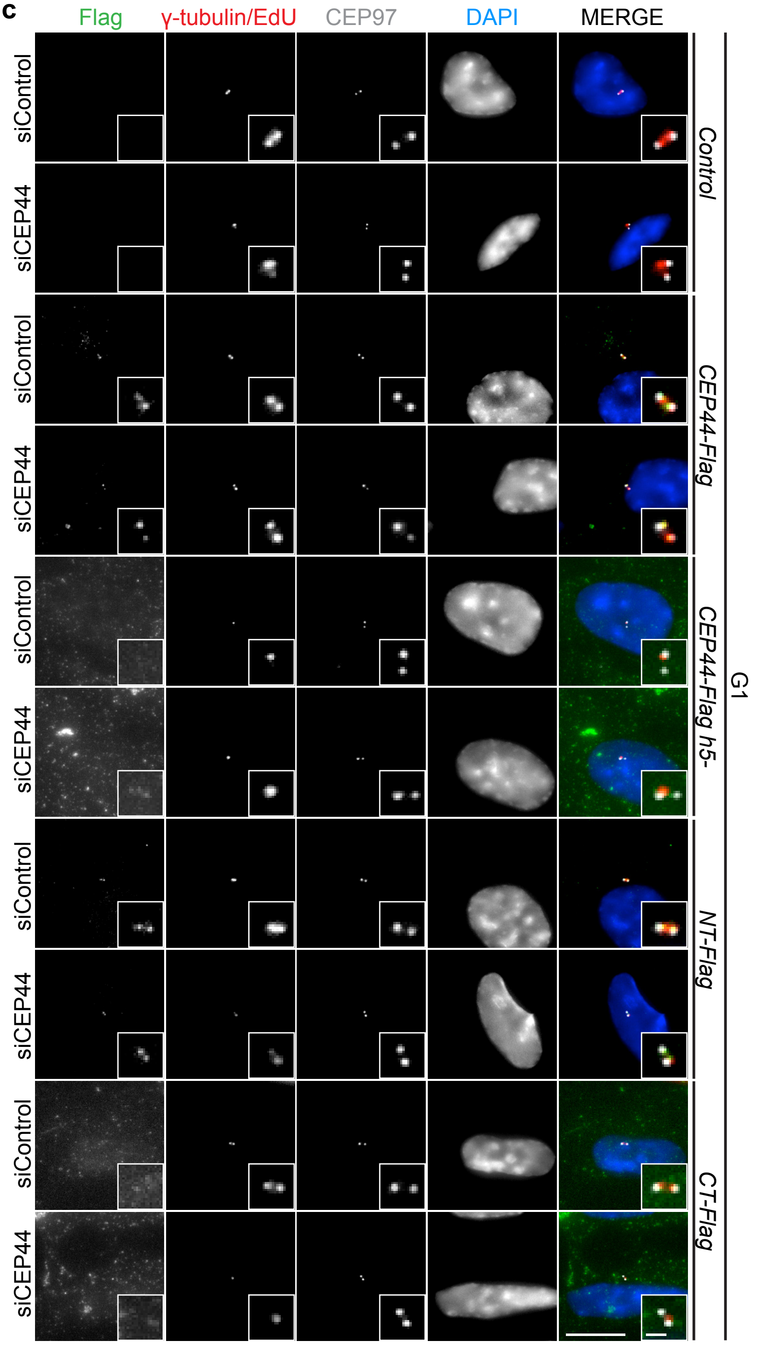

d

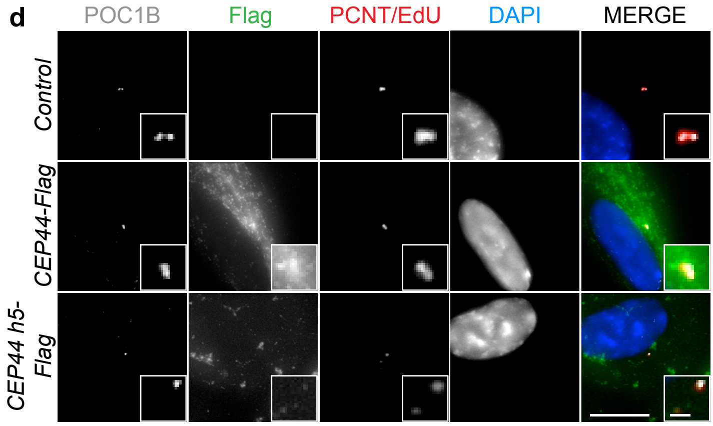

e

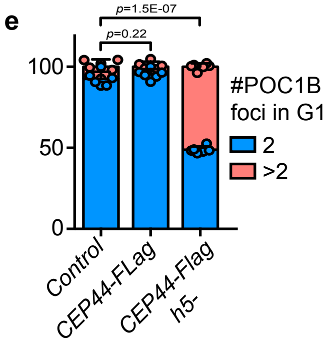

Supplementary figure 9. Mutations in CEP44 MT-binding domain generate a dominant negative conversion phenotype. Related to figure 4. (a) Summary of *CEP44* mutants used for the MT-binding study. The right column indicates the amino acidic changes in CEP44. The left column describes the position of the mutations (h=helix followed by the number of the helix, Fig. 4i). In the two upper mutants, positively charged amino acids were mutated to negative amino acids. In the following 4 constructs the folding of the helix was interrupted by mutating both leucine and isoleucine residues into proline. In the last four constructs, mutations were introduced to substitute the aliphatic amino acids into alanine in order to disrupt the side interactions between the helices. (b) IB of cell lysates to test expression of CEP44-Flag $h5^-$ in the mutant cell line in Fig. 4j and in (c). (c) IF of the samples in CEP44 rescue experiment described in (Fig. 4j and k). (d) POC1B delocalization from centrioles upon overexpression of the *CEP44* $h5^-$ mutant. (e) Quantification of (d). 51.2±2.0% of the cells expressing the *CEP44* $h5^-$ mutant lost POC1B from at least one of the centrosomes. Data are presented as mean±s.d. and statistics were derived from two-tail unpaired t-test analysis of n=6 biologically independent experiments and source data are provided as a Source Data file. (c, d, scale bars: 10 µm, magnification scale bars: 1 µm).

Supplementary figure 10. CEP295 accumulates on newly formed dCs. Related to figure 5

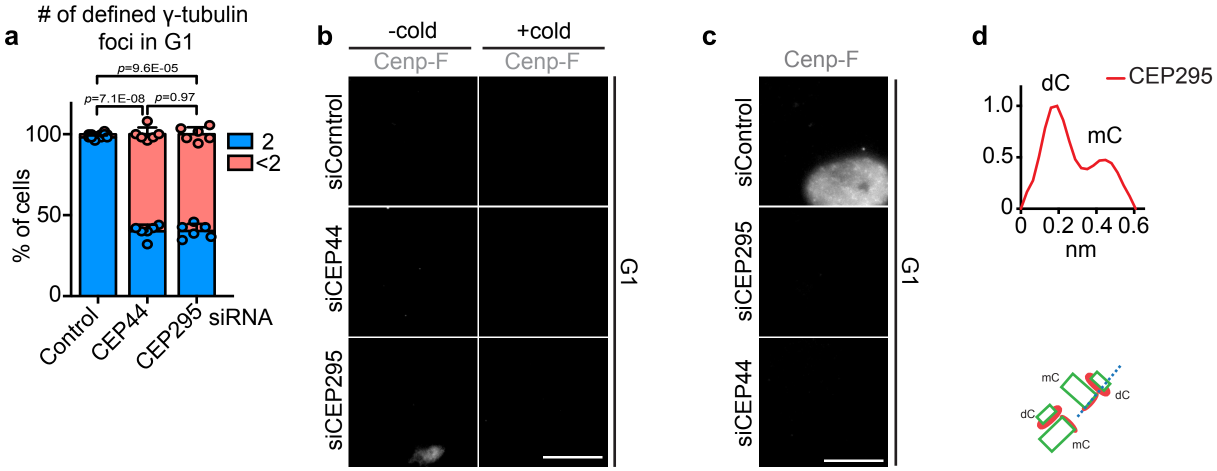

Supplementary figure 10. CEP295 accumulates on newly formed dCs. Related to figure 5. (a). In this CEP44 depletion condition 60.0±4.2% of G1 cells presented CCC defect as judged by γ-tubulin foci vs. siCEP295 sample in which 59.8±12.8% of G1 cells showed the CCC defect. Data are presented as mean±s.d. and statistics were derived from two-tail unpaired t-test analysis of n=6 biologically independent experiments and source data are provided as a Source Data file. (b) Cenp-F negative stain of G1 cells from Fig. 5a. (c) Cenp-F negative stain of G1 cells from Fig. 5c. (d) Normalized intensity plots profiles of mC (cross-section) and dC (longitudinal section) in Fig. 4f. CEP295 signal on dC is double so stronger than the one on the mC. (b and c, scale bars: 10 μm).

Supplementary figure 11. Centriole de-glutamylation does not play a role in CCC. Related to figure 6

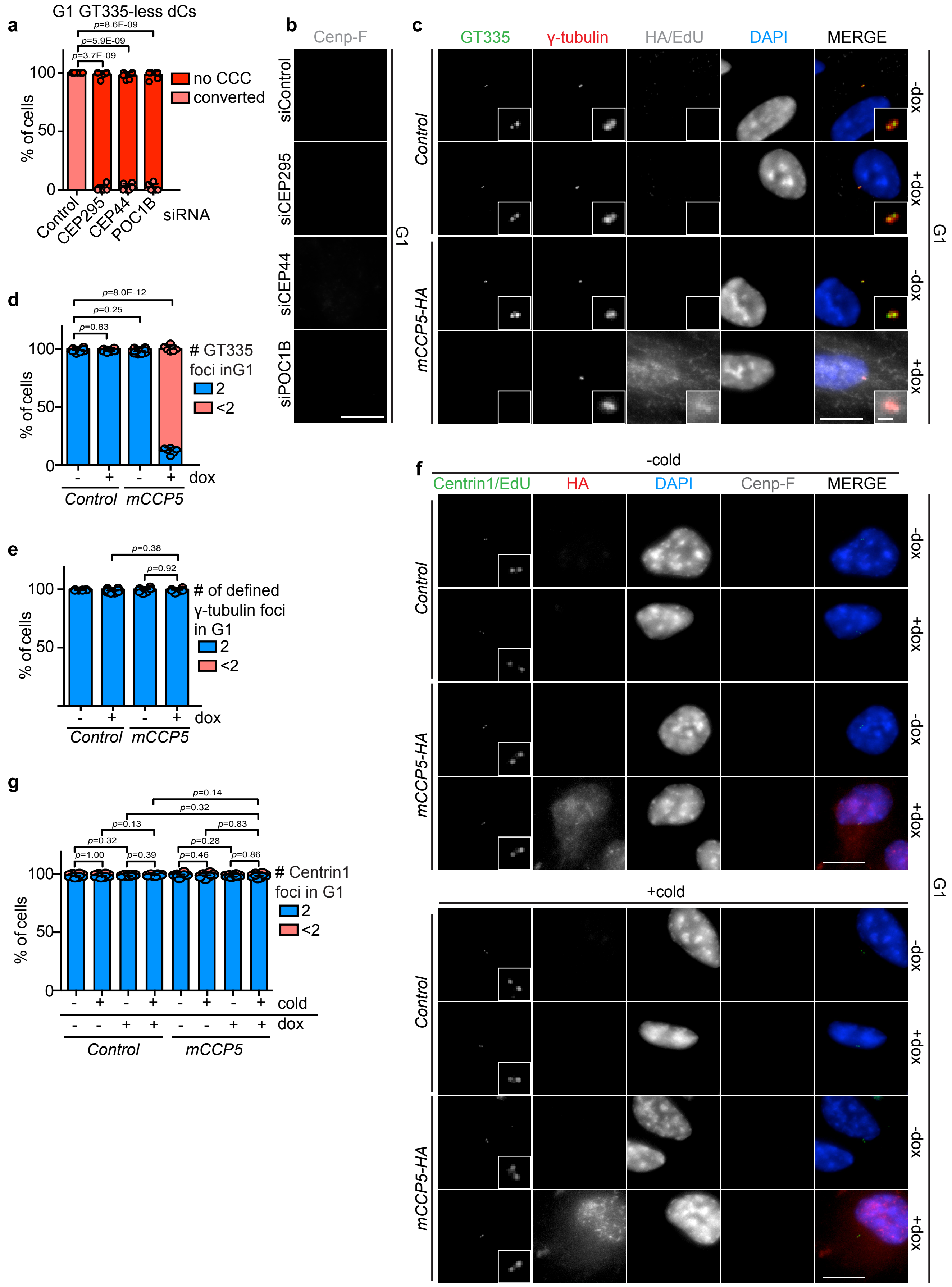

Supplementary figure 11. Centriole de-glutamylation does not play a role in CCC. Related to figure 6. (a) Quantification of de-glutamylated dCs from Fig. 6b that showed loss of PCNT and thus could not convert. In siCEP295 samples the loss of GT335 foci correlated in 98.7±2.8% of the cases with the loss of PCNT foci. In siCEP44 samples it correlated in 97.6±3.0% of the cases and in siPOC1B in 98.0±3.2%. Bars: SD. (b) Cenp-F negative stain of G1 cells from Fig. 6a. (c) Overproduced (1 µg/ml of doxycycline) mCCP-5-HA localized at the centrosome (HA signal). (d and e) mCCP-5-HA reduced in 87.7±2.8% of the G1 cells the glutamylation of centrioles (d), but did not affect the efficiency of dCs to recruit PCM (γ-tubulin) (e). (f) Centrioles exposed to mCCP5 as in (c and d) did not show any degree of instability (g) as judged by number of centrin1 foci per G1 cells. (g) Quantification of (f). (b, c, f, scale bars: 10 µm, magnification scale bars: 1 µm; a, d, e and g data are presented as mean±s.d., all statistics were derived from two-tail unpaired t-test analysis of n=6 biologically independent experiments and source data are provided as a Source Data file).

Supplementary figure 12. TUBD1- and TUBE1-less G1 centrioles do not undergo glutamylation. Related to figure 7

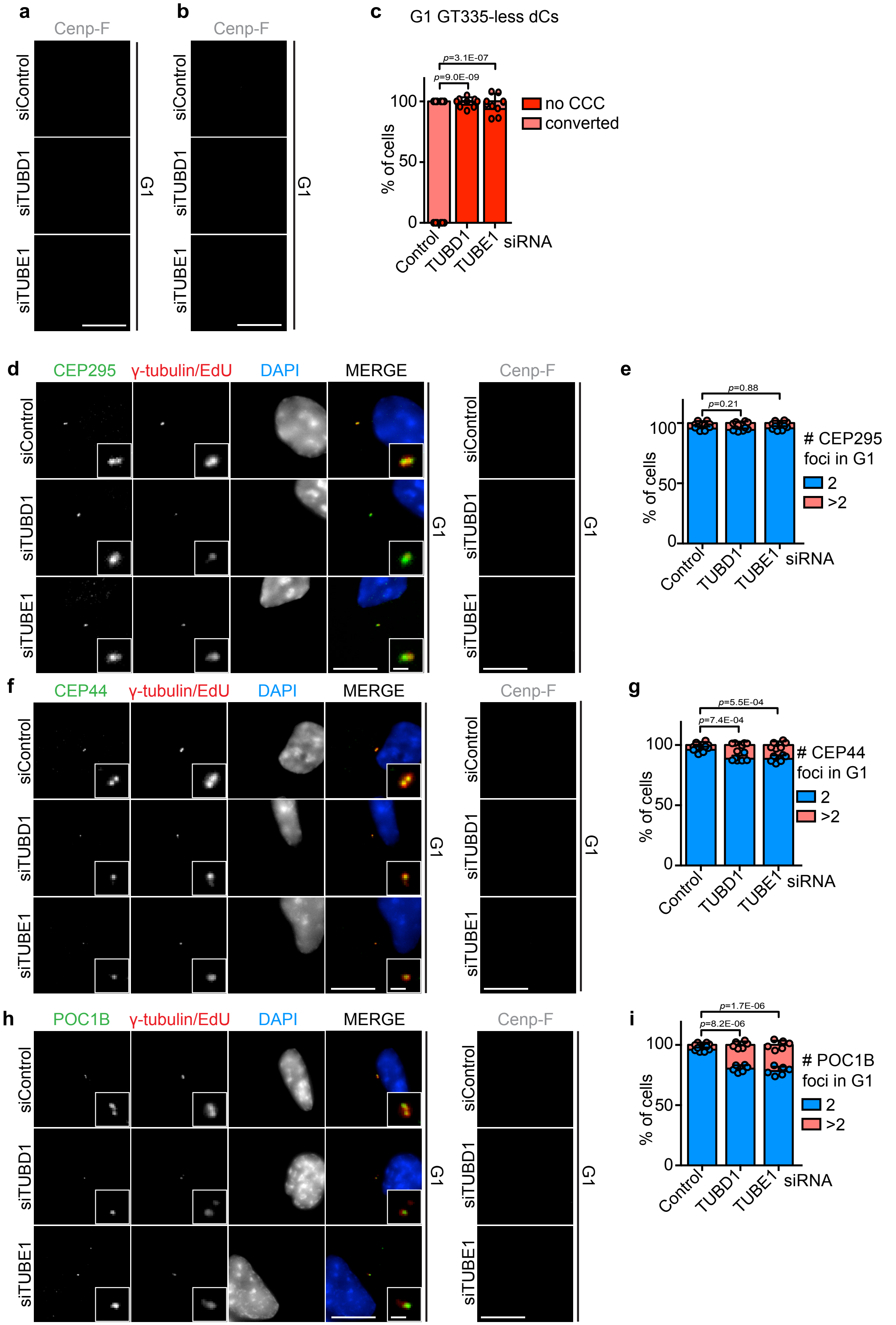

Supplementary figure 12. TUBD1- and TUBE1-less G1 centrioles do not undergo glutamylation. Related to figure 7. (a and b) Cenp-F negative stain of G1 cells respectively from Fig. 7d and f. (c) Quantification of de-glutamylated dCs from Fig. 7d that showed loss of PCNT and thus could not convert. In siTUBD1 samples the loss of GT335 foci correlated in 93.8±6.4% of the cases with the loss of PCNT foci and in siTUBE1 in 97.2±3.2% of the cases. (d) Depletion of either TUBD1 or TUBE1 did not delocalize CEP295. (e) Quantifications of (d). (f-i) Differently, depletion of TUBD1 and TUBE1 affected the localization of both CEP44 and POC1B. (g) Quantification of (f). CEP44 was delocalized in 21.4±2.7% of cells treated with siTUBD1 and in 21.5±2.9% of cells treated with siTUBE1. (i) Quantification of (h). POC1B was delocalized in 19.6±2.8% of cells treated with siTUBD1 and in 21.6±23.5 % of cells treated with siTUBE1. (a, b, d, f, h, scale bars: 10 µm, magnification scale bars: 1 µm; c, e, g and i data are presented as mean±s.d., all statistics were derived from two-tail unpaired t-test analysis of n=6 biologically independent experiments and source data are provided as a Source Data file).

Supplementary Table 1 - Mass spectrometry

Gene.names	Ratio Cep44-Flag/control	Ratio Cep44-Flag/control Normalized
POC1A	2.9123	2.7126
POC1B	2.2155	2.0595

Supplementary Table 2 - siRNAs/sgRNAs

siRNA		seq 5'-3'		cat#
siControl	ON-TARGET plus Non-targeting siRNA #1	UGGUUUACAUGUCGACUAA	Dharmacon	D-001810-01
siCEP44	ON-TARGET plus Human CEP44 siRNA - SMARTpool	CUGAAUAGGUCUCGGUAAA	Dharmacon	L-014639-01
		GGACUGUGUAGGUUUGAUA		
		CCAAUGUGGGUUUGCAGAA		
		AGUCUUAGCUGAAGGUAAA		
siCEP295	Silencer Select siKIAA1731	GUGAUACACUAACAAUUGA	Ambion	#4392420
siPOC1A	FlexiTube siRNA Hs_WDR51A_2	CUGGGUACCCAAUGUCAAA	QIAGEN	#SI03048871
siPOC1B	FlexiTube siRNA Hs_WDR51B_4	GAUUCCGUUGGAUUUGCAA		
siTUBD1	ON-TARGET plus Human TUBD1 siRNA - SMARTpool	GGAUUAGGAGCUUUCGUUA	Dharmacon	L-006843-00
		GAUCAGUACUCAAACUCAU		
		UUAUGGAACUGGUGAGGUU		
		CAGACGAAAUCCACUAGGA		
siTUBE1	ON-TARGET plus Human TUBE1 siRNA - SMARTpool	CGAGCAGUCUUGAUUGAUA	Dharmacon	L-006844-00
		UCAGAUCUUCGUAGAAAUA		
		GCACUCAUGGUUAGAGGAA		
		UUUCAGAUGCCUUUAGUAA		
sgRNA	exon1 Cas9	AAGCUUACGGAACCUAGAAC	cloned into pX458	

Supplementary Table 3 - Construct and plasmid list

**In vivo expression vector/construct (primers 5'->3')**	
pRetroX-TRE3G-CEP44-Flag	TCCATCGATACGCGTGCGCCACCATGGCAACAGGTGACTTAAAAAGA
	CCTCCCCTACCCGGTAGttaCTTGTCATCGTCGTCCTTGTAGTCTGCACCAGCTCCTGCACCTGCACCAGCTCCCAAGTAGTGATTTGGACATTTCAG
pRetroX-TRE3G-CEP44-Flag siRNA2 resistant	TTATCCTGAAGAGGTGGAtTGcGTgGGgctcATAAAGGGAGACCCAGC
	GCTGGGTCTCCCTTTATgagcCCcACgCAaTCCACCTCTTCAGGATAA
siRNA3 resistant	GACAAAAAAGCAGTTTATCCAgTGcGGaTTcGCcGAATGGAAAATCCAAATTGTTTG
	CAAACAATTTGGATTTTCCATTCgGCgAAtCCgCAcTGGATAAACTGCTTTTTTGTC
pRetroX-TRE3G-NT-CEP44 (1-195aa) -Flag	GATCCATCGATACGCGTGCGCCACCATGGCAACAGGTGACTTAAAAAG
	CCTCCCCTACCCGGTAGTTACTTGTCATCGTCGTCCTTGTAGTCTGCACCAGCTCCTGCACCTGCACCAGCTCCTGTTATTGGACTTAATGTATCCTCAG
pRetroX-TRE3G-CT-CEP44 (196-390aa)-Flag	GATCCATCGATACGCGTGCGCCACCATGGATGTTAATGAAGCAGTTGATG
	CCTCCCCTACCCGGTAGTTACTTGTCATCGTCGTCCTTGTAGTCTGCACCAGCTCCTGCACCTGCACCAGCTCCCAAGTAGTGATTTGGACATTTC
pRetroX-TRE3G-CEP44(1-310aa)-Flag	CATCGATACGCGTGCGCCACCATGGCAACAGGTGAC
	GGTAGAATTCGGGCCTTACTTGTCATCGTCGTCCTTGTAGTCTGCACCAGCTCCTGCACCAGCGTAGTCTTCACTAAC
pRetroX-TRE3G-CEP44(31-390aa)-Flag	CATCGATACGCGTGCGCCACCATGTTGATAAAGGGAGACC
	GGTAGAATTCGGGCCTTACTTGTCATCGTCGTCCTTGTAGTCTGCACCAGCTCCTGCACCCAAGTAGTGATTTGGACATTTC
pRetroX-TRE3G-CEP44(68-390aa)-Flag	CATCGATACGCGTGCGCCACCATGAAAAATGACTTGCGC
	GGTAGAATTCGGGCCTTACTTGTCATCGTCGTCCTTGTAGTCTGCACCAGCTCCTGCACCCAAGTAGTGATTTGGACATTTC
**pRetroX-TRE3G-CEP44 (h5-)-Flag (primers 5'->3')**	
K68D, R72E,K79D mutations	CCAATGTAGAGCTCATAGCAGATAATGACTTGGAATTTATAGATGCTGTCTATGACCTTCTTCGTGATCAATTTAATTATAAACC<br>TGCTATGAGCTCTACATTGG
R82D, K88D mutations	GATGCTGTCTATGACCTTCTTGATGATCAATTTAATTATGACCCAATTTTGACAAAAAAGCAG
	AAGAAGGTCATAGACAGCATC
pRetroX-TRE3G-POC1B-HA	GTCTTATACTTGGATCCATCGATAAATGGCCTCAGCCAC
	CCTGCACCTGCACCAGCTCCTGCGGCCGCGCTTTTCTGTTGGACAGC
pRetroX-TRE3G-mCCP5-HA	GATCCATCGATACGCGTGCATGGAGCTGCGCTG
	CCCGGTAGAATTCGGGCCTTAAGCGTAATCTGGAACATCGTATGGGTATGCACCAGCTCCTGCACCTGCACCAGCTCCTGCGGCCGCTCCCTCTGCGAGTC
**Protein purification vector/construct (primers 5'->3')**	
pGex-6P-1-GST-Flag	GTTCCAGGGGCCCCTGGGGATGGGAGCTGGTGCAGGTGCAGGAGCTGGTGCAGACTACAAGGACGACGATGACAAGTAACCCGGGTCGACTCGAGC<br>GCTCGAGTCGACCCGGGTTACTTGTCATCGTCGTCCTTGTAGTCTGCACCAGCTCCTGCACCTGCACCAGCTCCCATCCCCAGGGGCCCCTGGAAC
pGex-6P-1-CEP44-Flag	CTGTTCCAGGGGCCCCTGGCCGCAACAGGTGACTTAAAAAG
	CGCTCGAGTCGACCCGGGTTACTTGTCATCGTCGTCC
pGex-6P-1-CEP44 (h5-)-Flag	CTGTTCCAGGGGCCCCTGGccGCAACAGGTGACTTAAAAAG
	CGCTCGAGTCGACCCGGGTTACTTGTCATCGTCGTCC
pGex-6P-1-POC1B-HA	GTTCTGTTCCAGGGGCCCCTGGGGGCCTCAGCCACGGAGGAC
	CGGCCGCTCGAGTCGACCCGGGTTACTTGTCATCGTCGTCCTTGTAGTCTGCACCAGCTCCTGCAC
**Antibodies production vector/construct (primers 5'->3')**	
pET28c-CEP44	GCAAATGGGTCGGATCCGAATGGCAACAGGTG
	GTGCTCGAGTGCGGCCGCAagCAAGTAGTGATTTGGACATTTC
pGex-6P-1-POC1B(304-422aa)-HA	GGAAGTTCTGTTCCAGGGGCCCCTGGGGGAATTGCATTGTAAAGGTCTTAC
	CGATGCGGCCGCTCGAGTCGACCCGGGTTACCTTTGACTTTCACAGGG
pGex-6P-1-POC1A(296-369aa)-HA	GAAGTTCTGTTCCAGGGGCCCCTGGGGGTTTGGAAGAGTAACTTTGATATTG
	GATGCGGCCGCTCGAGTCGACCCGGGTTACACAATGTGCTCCAGC
CEP44 genome seq exon1	GACACCCTATATTCAGAAACTTATAG
	GGAATTCTTCATTAGTTCATACTTAG

Supplementary source data - Immunoblots 1

Related to Fig. 3a; anti-POC1B

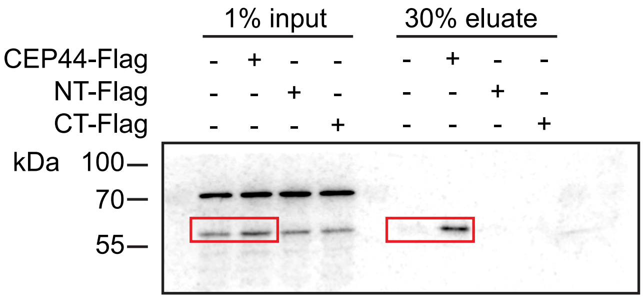

Related to Fig. 3a; anti-POC1A

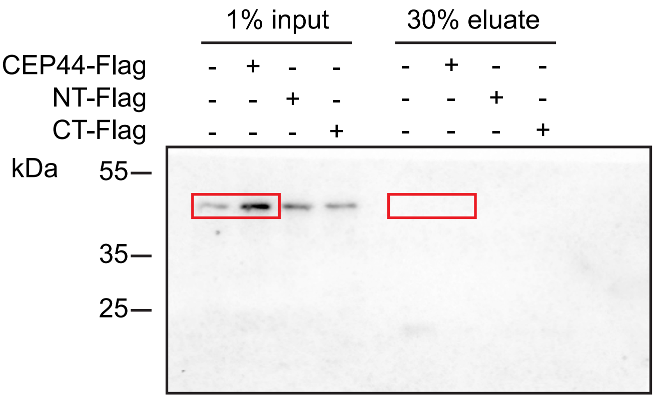

Related to Fig. 3a; anti-CEP295

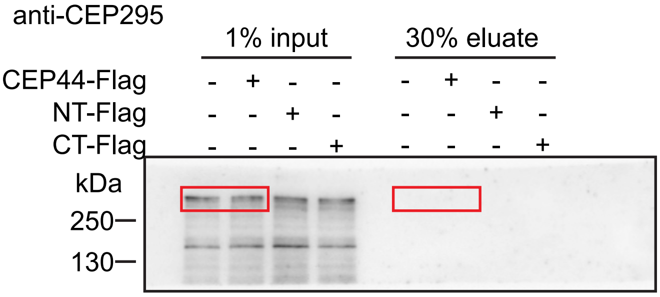

Related to Fig. 3a; anti-Flag

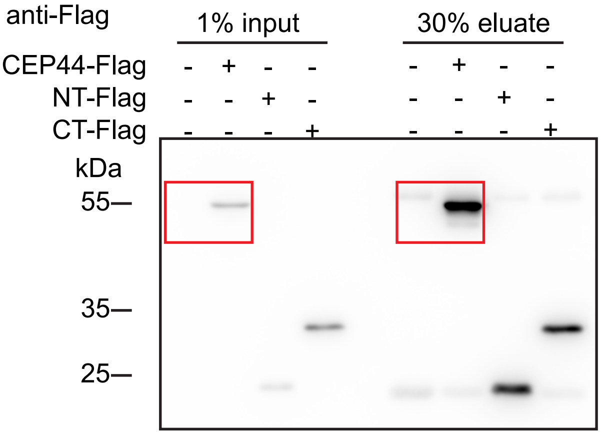

Related to Fig. 3a; anti-GAPDH

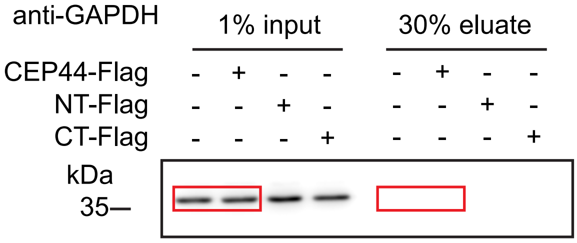

Related to Fig. 3e; anti-POC1B

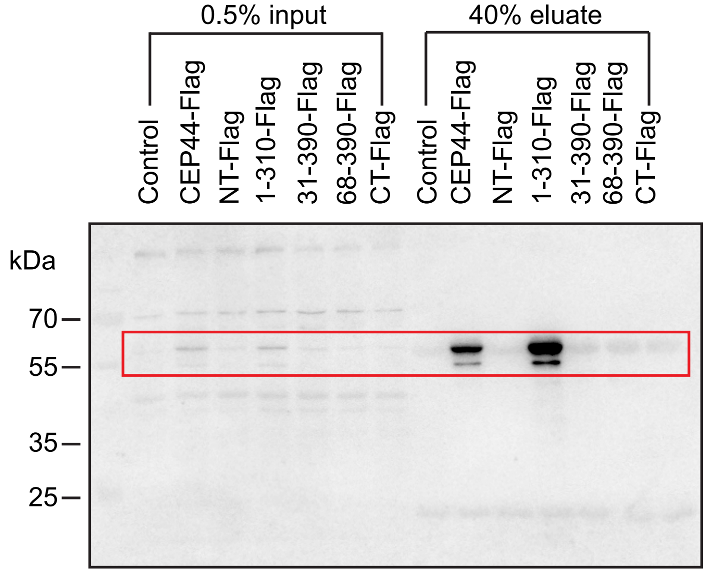

Related to Fig. 3e; anti-Flag

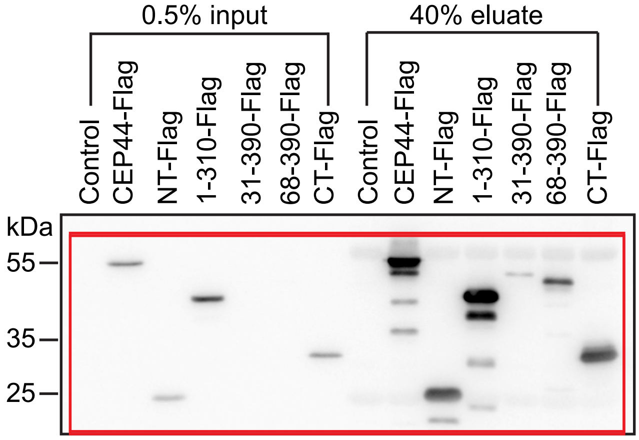

Supplementary source data - Immunoblots 2

Related to Fig. 3e; anti-GAPDH

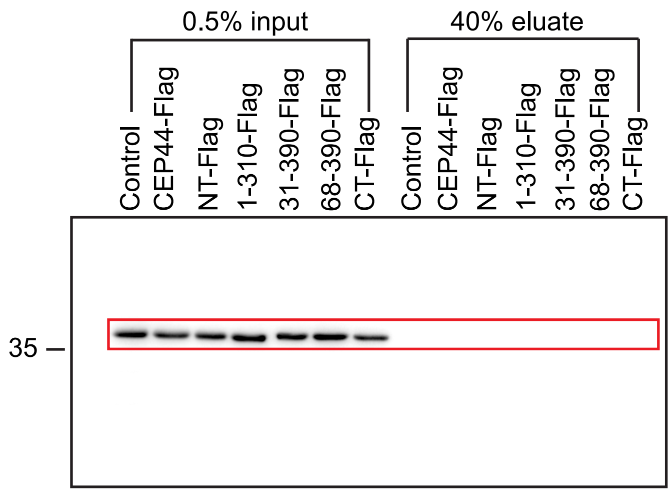

Related to Fig. 4h; anti-Flag

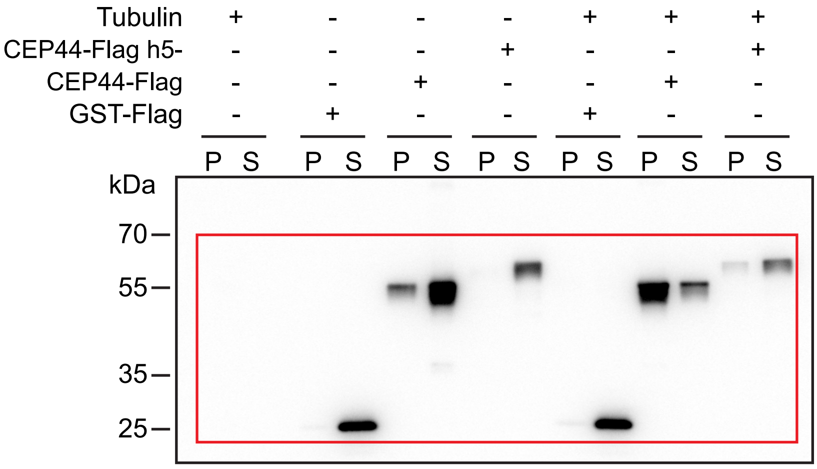

Related to Fig. 4h; anti-α-tubulin

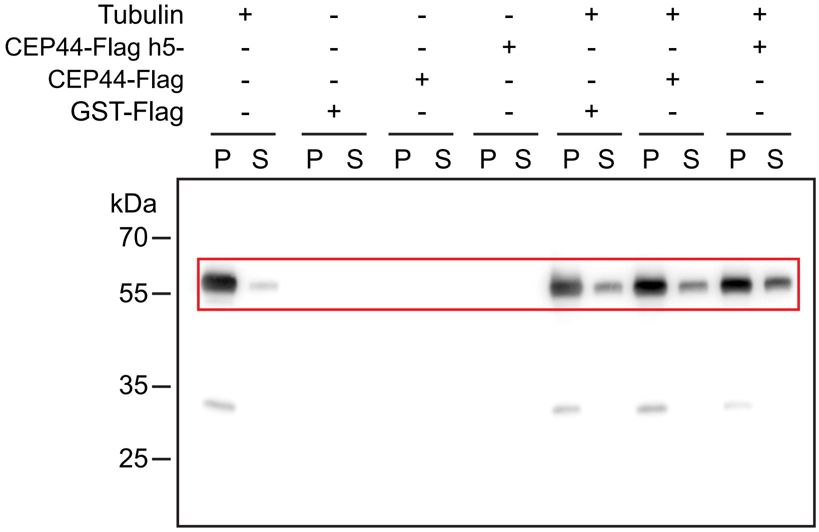

Related to Supplementary Fig. 1a; anti-CEP295

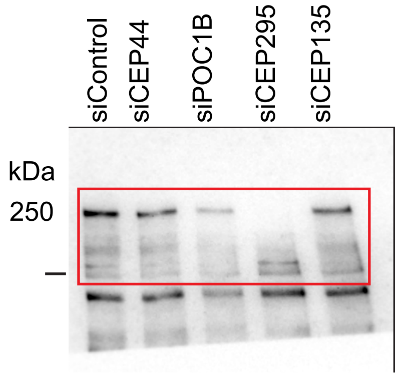

Related to Supplementary Fig. 1a; anti-GAPDH

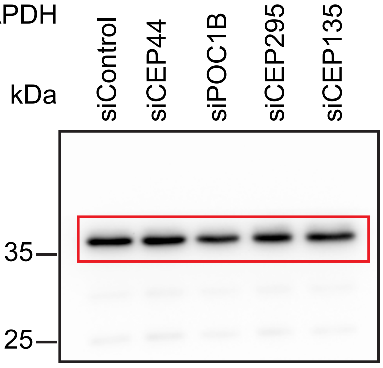

Related to Supplementary Fig. 1a; anti-CEP135

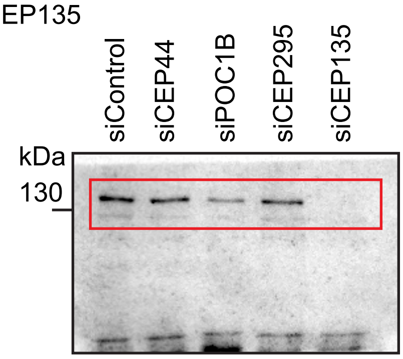

Related to Supplementary Fig. 1a; anti-CEP44

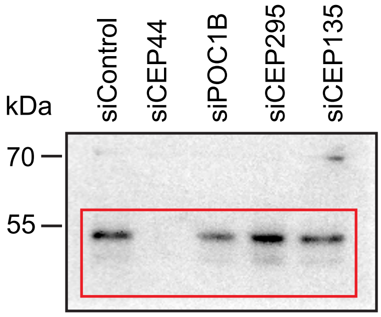

Related to Supplementary Fig. 1a; anti-POC1B

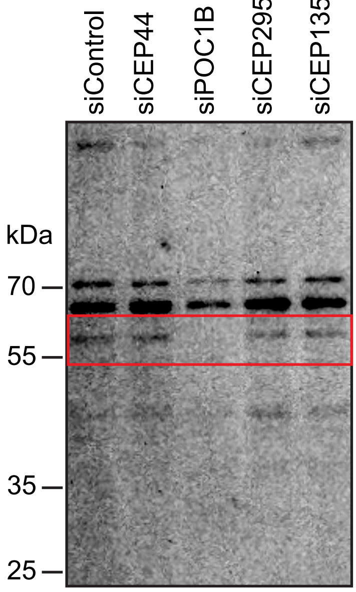

Supplementary source data - Immunoblots 3

Related to Supplementary Fig. 2b; anti-CEP44

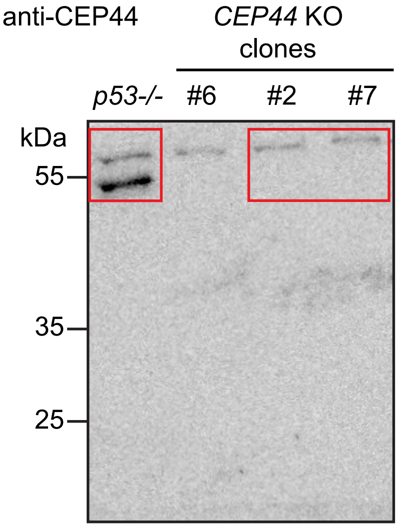

Related to Supplementary Fig. 2b; anti-RAD21

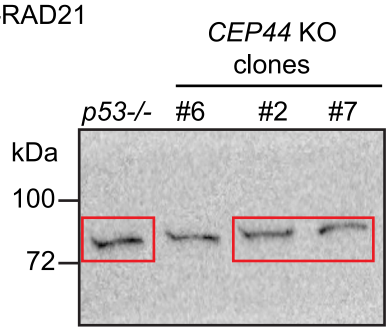

Related to Supplementary Fig. 3h; anti-CEP295

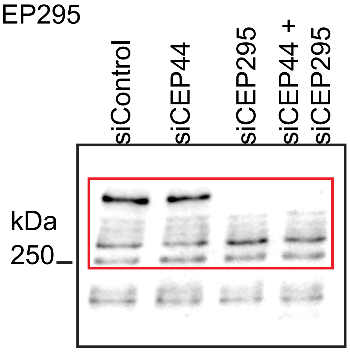

Related to Supplementary Fig. 3h; anti-CEP44

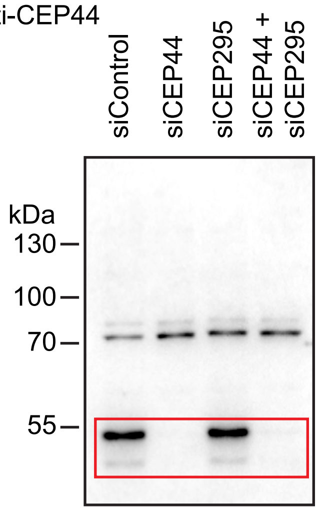

Related to Supplementary Fig. 3h; anti-GAPDH

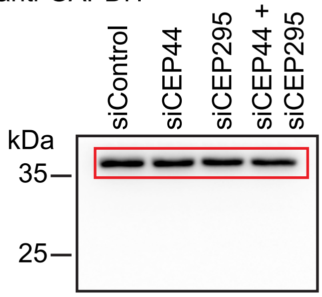

Related to Supplementary Fig. 5b; anti-HA

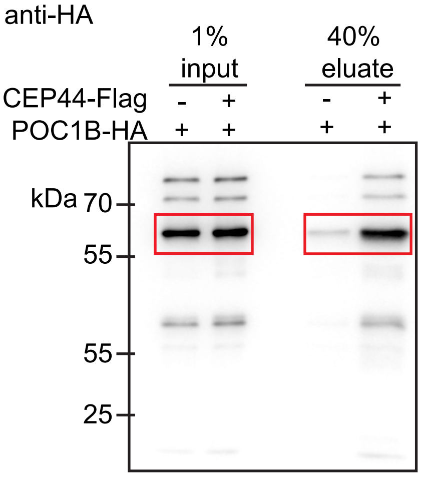

Related to Supplementary Fig. 5b; anti-Flag

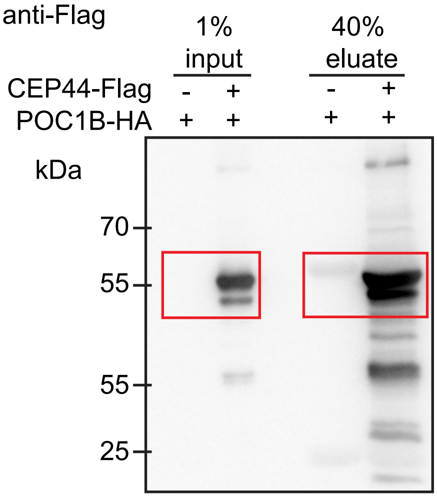

Related to Supplementary Fig. 9b; anti-Flag

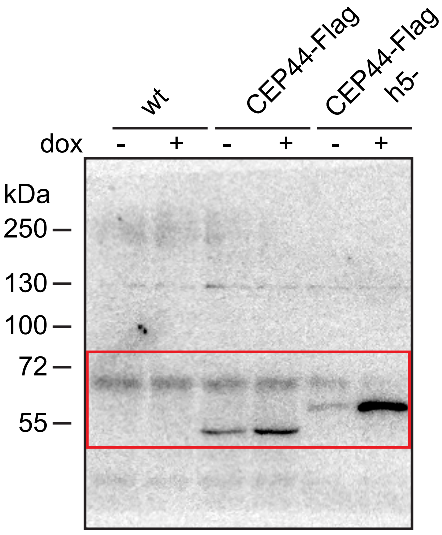

Related to Supplementary Fig. 9b; anti-GAPDH

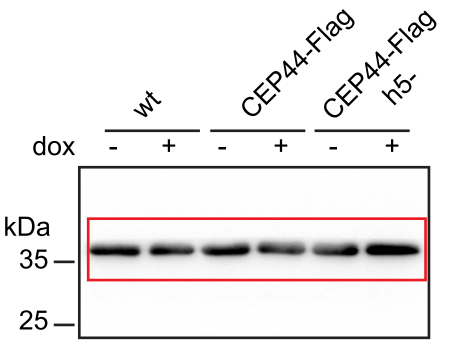

Supplement: Supplementary file 1 — Supplementary Information [file 41467_2020_14767_MOESM1_ESM.pdf]
