# Supplementary material for: CEP44 ensures the formation of bona fide centriole wall, a requirement for the centriole-to-centrosome conversion
Source: Nat Commun. 2020 Feb 14;11:903. doi: 10.1038/s41467-020-14767-2 (PMC7021698; doi:10.1038/s41467-020-14767-2)
Supplement: Supplementary file 3 — Reporting Summary [file 41467_2020_14767_MOESM3_ESM.pdf]

nature research

Corresponding author(s): Elmar Schiebel

Last updated by author(s): Jan 21, 2020

Reporting Summary

Nature Research wishes to improve the reproducibility of the work that we publish. This form provides structure for consistency and transparency in reporting. For further information on Nature Research policies, see Authors & Referees and the Editorial Policy Checklist .

Statistics

For all statistical analyses, confirm that the following items are present in the figure legend, table legend, main text, or Methods section.

n/a	Confirmed	
☐	☒	The exact sample size (n) for each experimental group/condition, given as a discrete number and unit of measurement
☐	☒	A statement on whether measurements were taken from distinct samples or whether the same sample was measured repeatedly
☐	☒	The statistical test(s) used AND whether they are one- or two-sided<br>*Only common tests should be described solely by name; describe more complex techniques in the Methods section.*
☐	☒	A description of all covariates tested
☐	☒	A description of any assumptions or corrections, such as tests of normality and adjustment for multiple comparisons
☐	☒	A full description of the statistical parameters including central tendency (e.g. means) or other basic estimates (e.g. regression coefficient) AND variation (e.g. standard deviation) or associated estimates of uncertainty (e.g. confidence intervals)
☐	☒	For null hypothesis testing, the test statistic (e.g. F, t, r) with confidence intervals, effect sizes, degrees of freedom and P value noted<br>*Give P values as exact values whenever suitable.*
☒	☐	For Bayesian analysis, information on the choice of priors and Markov chain Monte Carlo settings
☒	☐	For hierarchical and complex designs, identification of the appropriate level for tests and full reporting of outcomes
☒	☐	Estimates of effect sizes (e.g. Cohen's d, Pearson's r), indicating how they were calculated

Our web collection on statistics for biologists contains articles on many of the points above.

Software and code

Policy information about availability of computer code

Data collection	Immuno-Fluorescence data were acquired with softWoRx v6.1.1 Release 5 (Applied Precision, GE), 2D-SIM data using NIS-Elements software v4.6 (Nikon), Immuno-blots and Coomassie Blue stained gels were acquired with LAS4000IR v2.1 software and Electron Microscopy images using SerialEM (v3.7)
Data analysis	Immuno-fluorescence, 2D-SIM, Immuno-blots, Coomassie Blue stained and Electron Microscopy data were analysed by Fiji (ImageJ v2.0.0-rc-30/1.49t). GraphPad Prism v6 (GraphPad Software) was used for the data representation and mean and standard deviation values calculation. Microsoft Excel was used for p-values calculation, data pools normalization and generation of intensities plot profiles.

For manuscripts utilizing custom algorithms or software that are central to the research but not yet described in published literature, software must be made available to editors/reviewers. We strongly encourage code deposition in a community repository (e.g. GitHub). See the Nature Research guidelines for submitting code & software for further information.

Data

Policy information about availability of data

All manuscripts must include a data availability statement. This statement should provide the following information, where applicable:

- Accession codes, unique identifiers, or web links for publicly available datasets
- A list of figures that have associated raw data
- A description of any restrictions on data availability

All data that support the findings of this study are available in the Source Data and Supplementary Information files.

Field-specific reporting

Please select the one below that is the best fit for your research. If you are not sure, read the appropriate sections before making your selection.

- [x] Life sciences
- [ ] Behavioural & social sciences
- [ ] Ecological, evolutionary & environmental sciences

For a reference copy of the document with all sections, see nature.com/documents/nr-reporting-summary-flat.pdf

Life sciences study design

All studies must disclose on these points even when the disclosure is negative.

Sample size	No statistical methods were used to predetermine sample size. Data were collected to have a sufficient number of cells/centrosome/centrioles for statistic analysis. Sample size was chosen based on data variation.
Data exclusions	No data were excluded from the analyses.
Replication	Immunoprecipitation, mass spectrometry, immuno-blots, Coomassie blue stained gels, cell treatments, were repeated at least three times to confirm the reproducibility of the experiment. All experiments were successful replicated.
Randomization	Immuno-fluorescence data were acquired randomly . In vitro studies were not related to randomization.
Blinding	For immuno-fluorescence experiments it was not feasible to apply blinding experiments beacause of large sample sizeas well as for Electron Microscopy experiments. In other cases, the findings were confirmed by independent approaches.

Reporting for specific materials, systems and methods

We require information from authors about some types of materials, experimental systems and methods used in many studies. Here, indicate whether each material, system or method listed is relevant to your study. If you are not sure if a list item applies to your research, read the appropriate section before selecting a response.

Materials & experimental systems

n/a	Involved in the study	
[ ]	[x]	Antibodies
[ ]	[x]	Eukaryotic cell lines
[x]	[ ]	Palaeontology
[x]	[ ]	Animals and other organisms
[x]	[ ]	Human research participants
[x]	[ ]	Clinical data

Methods

n/a	Involved in the study	
[x]	[ ]	ChIP-seq
[x]	[ ]	Flow cytometry
[x]	[ ]	MRI-based neuroimaging

Antibodies

Antibodies used

CEP44 antibodies were raised in rabbits immunized against the CEP44 6xHis-CEP44 POC1A and used 1:200 for IF and 1:100 for IB. POC1A antibodies were raised in guinea pigs immunized against the peptide 296-369aa and used 1:50 for IF. POC1B antibodies were raised in guinea pigs immunized against the peptide 304-422aa peptides and used 1:500 for IF and 1:100 for IB. See Methods section for further information. γ-tubulin antibodies were raised in guinea pigs immunized against the humn γ-tubulin-myc-his and used 1:50 for IF.

Antibodies against: γ-tubulin (mouse, 1:1000, abcam Ab27074 LOT#GR317345-16), PCNT (rabbit, 1:2000, abcam Ab4448 LOT#GR3200989-1), CEP97 (rabbit, 1:300, Bethyl A301-945A LOT#n.s.), Centrin1 (mouse 1:1000, Millipore MABC544 LOT#2872235), α-tubulin (mouse, 1:500, SigmaAldrich DM1A LOT#047M4789V), SASS-6 (mouse, 1:50, SCBT sc-81431 LOT#C0613), Flag tag (mouse, IF 1:1600 - WB 1:1000, Cell signaling 9A3 LOT#3), CEP295 (rabbit, 1:500, Abcam Ab122490 LOT#GR259105-2), GAPDH (rabbit, WB 1:1000, 14C10 LOT#10), HA tag (rat, 1:1000, Sigma Aldrich 11867431001), GT335 (mouse, 1:500, AdipoGen AG-2013-0020), CEP135 (rabbit, 1:100), C-Nap1 (goat, 1:1000), CEP164 (rat, 1:2000), CEP152 (rabbit, 1:500), CEP192 (rabbit, 1:2000) and Cenp-F (sheep, 1:1000)

Validation

CEP44 antibodies were validated by IF and IB of cells depleted with siCEP44 and in CEP44KO cell lines. Details are described in this study. POC1A and POC1B were validated by IF cells treated with either siPOC1A or siPOC1B. POC1A antobodies were validated also in IB by pilot experiments before using. POC1B antobodies were validated also in IB as described in this study. Home made γ-tubulin guinea pig antibodies were validated by pilot experiments before using.

Anti-γ-tubulin (mouse, 1:1000, abcam Ab27074 LOT#GR317345-16):

https://www.abcam.com/gamma-tubulin-antibody-tu-30-ab27074.html

Anti-PCNT (rabbit, 1:2000, abcam Ab4448 LOT#GR3200989-1):

https://www.abcam.com/pericentrin-antibody-centrosome-marker-ab4448.html

Anti-CEP97 (rabbit, 1:300, Bethyl A301-945A LOT#n.s.):

https://www.bethyl.com/product/A301-945A/CEP97+Antibody
Anti-Centrin1 (mouse 1:1000, Millipore MABC544 LOT#2872235):
http://www.merckmillipore.com/DE/de/product/Anti-Centrin-Antibody-clone-20H5,MM_NF-04-1624?ReferrerURL=https%3A%2F%2Fwww.google.com%2F&bd=1
Anti-α-tubulin (mouse, 1:500, SigmaAldrich DM1A LOT#047M4789V):
https://www.sigmaaldrich.com/catalog/product/sigma/t9026?lang=de&region=DE
Anti-SASS-6 (mouse, 1:50, SCBT sc-81431 LOT#C0613):
https://www.scbt.com/p/sas-6-antibody-91-390-21
Anti-Flag tag (mouse, IF 1:1600 - WB 1:1000, Cell signaling 9A3 LOT#3):
https://en.cellsignal.de/products/primary-antibodies/dykddddk-tag-9a3-mouse-mab-binds-to-same-epitope-as-sigma-s-anti-flag-m2-antibody/8146
Anti-CEP295 (rabbit, 1:500, Abcam Ab122490 LOT#GR259105-2):
https://www.abcam.com/cep295-antibody-ab122490.html
Anti-https://en.cellsignal.de/products/primary-antibodies/gapdh-14c10-rabbit-mab/2118
Anti-HA tag (rat, 1:1000, Sigma Aldrich 11867431001):
https://www.sigmaaldrich.com/catalog/product/roche/roahaha?lang=de&region=DE&gclid=EAIaIQobChMI0KTxtoCL5wIVS9HeCh32jwDnEAAYASAAEgJI1fD_BwE
GT335 (mouse, 1:500, AdipoGen AG-2013-0020):
https://adipogen.com/ag-20b-0020-anti-polyglutamylation-modification-mab-gt335.html
CEP135 (rabbit, 1:100, previously described in doi: 10.1242/jcs.179713)
C-Nap1 (goat, 1:1000 previously described in https://doi.org/10.1371/journal.pgen.1005243)
CEP164 (rat, 1:2000 described previously in doi: 10.1083/jcb.201202126)
CEP152 (rabbit, 1:500, previously described in https://doi.org/10.1083/jcb.201007107)
CEP192 (rabbit, 1:2000, previously descibed in doi: 10.1016/j.cub.2009.05.016)
Cenp-F (sheep, 1:1000, previously described in J Cell Sci. 2002 Sep 1;115(Pt 17):3403-14)

Eukaryotic cell lines

Policy information about cell lines

Cell line source(s)	RPE1, RPE1 C-Nap1 KO and HE293 were described in Panic, M. et al. PLOS Genet. 11, e1005243 (2015). HEK GP2-293 was described in Hata S. et al. Nature Cell Biology 21, 1138–1151 (2019). U2OS tetracyclin -inducible cell-line expressing Myc-PLK4 was described by Kleylein-Sohn J. et al. Dev. Cell 13(2):190-202 (2007). RPE1 p53 KO was described by Izquierdo, D. et al. Cell Rep. 8, 957–65 (2014).
Authentication	Cell lines were examined for their morphology by microscopy.RPE1 p53Ko was tested by immuno-blot. U2OS expressing Myc-PLK4 was tested by insert expression induction by immuno-fluorescence.
Mycoplasma contamination	All the cell lines were tested for mycoplasma contamination and found negative.
Commonly misidentified lines (See ICLAC register)	No commonly used misidentified cell lines were used in this study.
